# Absorbance summation: A novel approach for analyzing high-throughput ELISA data in the absence of a standard

**DOI:** 10.1371/journal.pone.0198528

**Published:** 2018-06-08

**Authors:** Holly Hartman, Yuge Wang, Harry W. Schroeder, Xiangqin Cui

**Affiliations:** 1 Department of Biostatistics, University of Alabama at Birmingham, Birmingham, Alabama, United States of America; 2 Department of Microbiology, University of Alabama at Birmingham, Birmingham, Alabama, United States of America; 3 Department of Medicine, University of Alabama at Birmingham, Birmingham, Alabama, United States of America; University of California, Davis, UNITED STATES

## Abstract

We have developed a very simple method, termed absorbance summation (AS), for comparing protein concentrations between samples in ELISA assays without a standard. This method sums the observed absorbance values from all dilutions to obtain one data point for each sample to be used for comparison. AS is less computationally intensive than fitting sigmoidal curves, and it avoids the difficulty of parameter estimation for samples with absorbance values lying primarily at the lower tail of the curve. Our simulation studies showed that it performs much better than the sigmoidal curve fitting method and the conventional endpoint titer method. The power of this simple method is as high as the formal curve fitting followed by the estimation of area under the curve (AUC).

## Introduction

ELISA (Enzyme-Linked ImmunoSorbent Assay) is a commonly used method in biomedical fields to determine the concentration of protein (analyte) in solution based on antigen-antibody binding. The amount of binding is reflected by the intensity of color change measured by light absorbance. When a standard absorbance curve is available, the concentration of the protein in sample can be estimated by comparing the sample absorbance and the standard curve. In cases where a standard is not available, the differences of protein concentrations of multiple samples are often compared based on serial dilutions.

There are two commonly used data analysis methods for comparing ELISA samples using serial dilutions in the absence of a standard. One is to fit a sigmoidal curve for each sample and then compare the estimated key parameter, points of maximum growth (PMG). Rodbard and Hutt [1] were the first to propose the use of a 4-parameter sigmoidal curve to analyze ELISA data. This method has been recommended as the most appropriate method for ELISA data [2]. When a sample has higher concentration of protein to be measured, all dilutions of this sample have higher absorbance, the sigmoidal curve shifts towards more diluted end. The drawback of this method is that fitting a 4-parameter curve is computationally rigorous, and thus it relies on having sufficient data for estimating the curve. When samples have very low concentration of protein to start with, it may not be possible to obtain accurate estimates for the sigmoidal curve parameters [3].

The second commonly used method is Endpoint Titer (ET). This method simply selects a cutoff absorbance level above the noise background. The highest dilution (or titer) that is above this cutoff level is the endpoint titer, which is used for further sample comparisons. This method is widely used due to its simplicity, since there is no need for computation. The drawback of this method is that it produces discrete data as the dilutions only take on a limited number of specific values. As a result, the most commonly used statistical approaches, such as t tests or ANOVA, cannot be used without violating key assumptions such as continuity or normality.

In this paper, we propose a simple alternative method that can be used for comparing protein concentrations between ELISA samples. We name this method absorbance summation (AS). The absorbance summation method overcomes the complexity of fitting sigmoidal curves and the difficulty of parameter estimation for samples with observed dat lying primarily at the lower tail of the curve. The AS method also avoids the disadvantage of discrete data that complicates the downstream analysis of ET method, while still achieving the simplicity of the ET method.

## Methods

### HIV gp140 binding dataset

The ELISA data used for analysis were obtained from Wang et al. and have been previously published [4]. This dataset was generated for studying the binding profiles of HIV gp140 epitope-binding antibodies in the sera of wild type and D altered mice. The third complementarity determining region of the immunoglobulin heavy (H) chain (CDR-H3) is the direct product of VDJ rearrangement and the focus of the diversity of the pre-immune antibody repertoire [5, 6]. Although in theory each diversity (D_H_) gene segment can be rearranged into one of six different reading frames (RF), three by deletion and three by inversion; in practice preference is observed for just one, RF1. RF1 is enriched for tyrosine and other neutral amino acids, inverted RF1 is enriched for arginine and other charged amino acids, and the four remaining D_H_ are enriched for hydrophobic amino acids. Coupled with control of reading frame usage, these preferences have the effect of biasing the amino acid profile of CDR-H3, which is not only the most diverse portion of the antibody, but also the portion of the antibody most frequently in contact with antigen [7, 8].

To test whether evolutionary constraints on D_H_ amino acid content, and thus on CDR-H3, influence epitope recognition, a series of mouse strains were created wherein the D_H_ locus had been simplified by using gene targeting techniques to delete 12 of the 13 D_H_ gene segments, and then either keeping or frameshifting the remaining D_H_ to bias for use of hydrophobic RF2 or charged inverted RF1 in place of neutral, tyrosine enriched RF1. B cells in these mouse strains produce a CDR-H3 repertoire that is enriched for tyrosine when either a wild type D_H_ locus or a single wild type D_H_ is used, or enriched for valine or arginine in place of tyrosine when either RF2 or inverted RF1 is emphasized. Four mouse strains with gp140 from HIV-1 JR-FL isolate were serially immunized and then assessed using ELISA for antibody binding to a panel of neutralizing or immunodominant HIV-1 envelope epitopes [4, 7].

Using ELISA, absorbance was measured for two different types of antibodies (IgG and IgM) from the four different mouse strains against 6 different antigen epitopes. A total of ten mice were used for each combination. Each mouse was immunized on five sequential occasions, and then bled 10 days after challenge. There were 12 different dilutions used in ELISA (1/30, 1/90, 1/270, 1/810, 1/2430, 1/7290, 1/21870, 1/65610, 1/196830, 1/590490, 1/1771470, and 1/5314410) to measure antibody concentrations. In order for the dilutions to be equidistant and since the dilutions are 3-fold, a log_3_ transformation of the dilutions was used throughout the analyses.

### Methods for analyzing ELISA data

**Fig 1:**
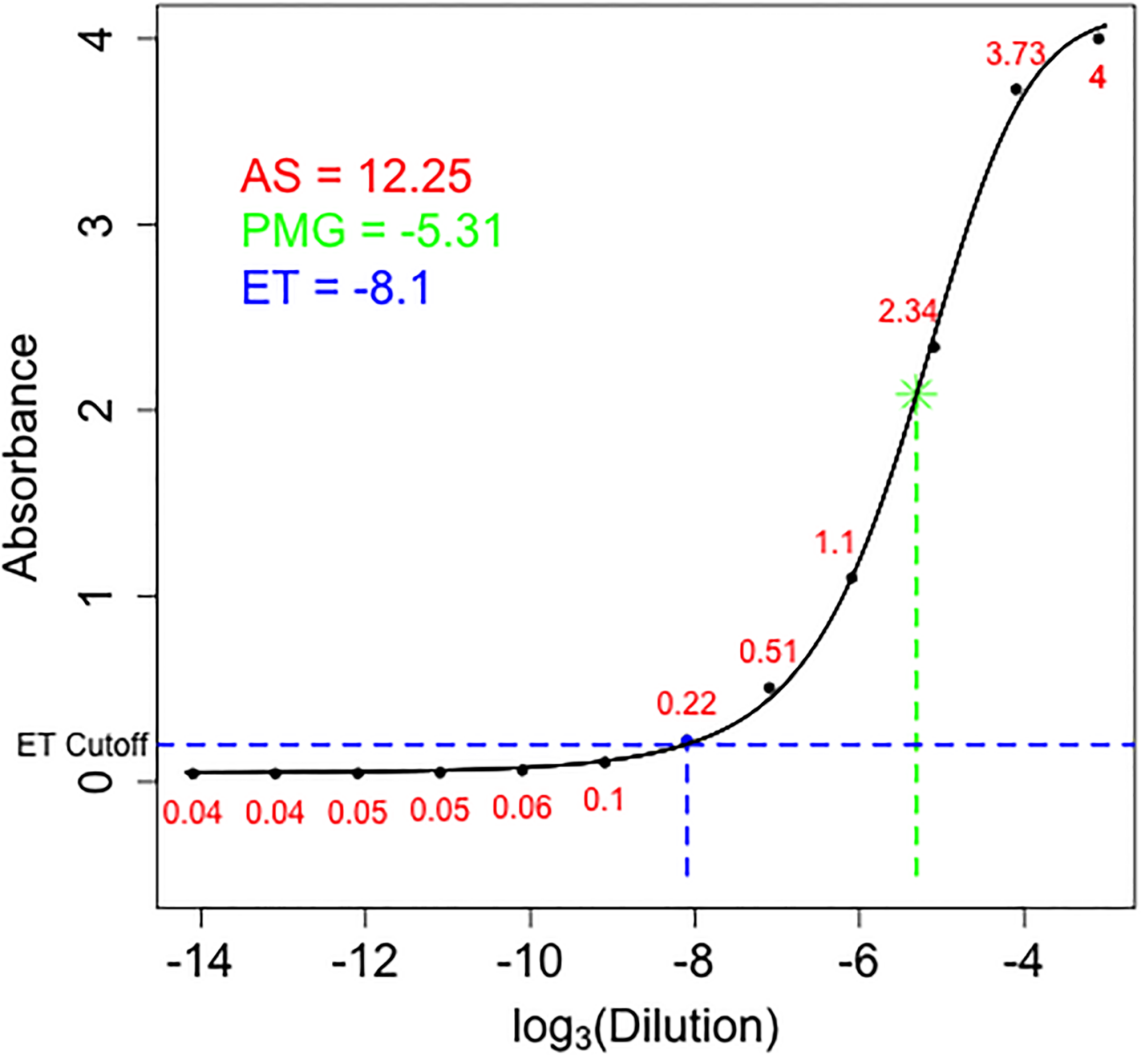
Example for summarizing the ELISA dilution absorbance data using three methods. The points are absorbance values (red numbers) at different dilutions from a sample in the dataset. The sigmoidal curve is a fitted 4-parameter model with the point of maximum growth shown as green *. The endpoint titer value is represented by the blue point above the blue dashed horizontal line. The absorbance summation is the sum of all the red numbers.

We use an example to illustrate the three methods in summarizing the ELISA dilution data (Fig 1).

#### Fitting sigmoidal models

Traditionally, a 4- or 5-parameter sigmoidal model has been used to fit ELISA data. The 4-parameter model we used is as follows:

$$f(x)\;=\;\frac{a-d}{1+{\left(\frac{x}{c}\right)}^{b}}+d$$ (1)

where *f*(*x*) is the resulting absorbance at dilution *x*; *a* is the maximum absorbance; *d* is the minimum absorbance; *c* is the point of maximum growth (or inflection point); *b* is the slope at *c*. To fit this 4-parameter sigmoidal model, we used the *nlsLM* function in the *minipack*.*lm* package in R (version 3.1.0). We used the log_3_ transformed dilution factors and the raw observed absorbance at each dilution. Errors are assumed to be normally distributed and equally weighted. All four parameters were estimated and the estimated *c* parameter is the parameter of interest, point of maximum growth (PMG). In the Fig 1 example, the fitted line provides a PMG of -5.31, the log3(Dilution) value for the green *.

#### Endpoint titer (ET) method

The endpoint titer method selects an absorbance value slightly above than the background noise (or the lower asymptotic level). The maximum dilution that has an absorbance greater than this preselected value is defined as the endpoint titer. The intuition behind this method is that a sample with more concentrated protein will need a higher dilution factor to have an absorbance just above background noise level. Traditionally an absorbance cutoff of 0.2 is used, however alternative methods have been proposed [9]. For the ET method, we used the conventional cutoff value of 0.2. In our Fig 1 example, the dilution that gives absorbance right above the 0.2 horizontal line corresponds to -8.1 on the x axis and has an absorbance of 0.22. We then set -8.1 to be the ET value for this sample.

#### Absorbance summation (AS) method

Since absorbance is measured at each dilution of a sample, our proposed method simply sums all the absorbance values from the same sample to obtain one value. We termed this value the absorbance summation (AS). In the Fig 1 example, the AS value is calculated as 0.04 + 0.04 + 0.05 + 0.05 + 0.06 + 0.1 + 0.22 + 0.51 + 1.1 + 2.34 + 3.73 + 4 = 12.25.

### Analyzing HIV gp140 binding data

The three methods described above were used to analyze the HIV gp140 binding ELISA data [4]. The dataset included 2,879 samples. However, a sigmoidal curve was estimable only for 1027 of these samples. All further method comparisons using this dataset were only conducted on these 1027 samples.

### Simulating ELISA data

As in the HIV gp140 binding dataset [4], ELISA absorbance readings were simulated using equidistant log_3_ dilutions and simulated data using the 4-parameter sigmoidal model in Eq 1. To avoid errors in parameter estimates at extreme values, we subtracted 10 from the log_3_ dilutions. Our final dilution values were from -24.0959 to -13.0959, increasing by 1. We set *a* as 4, *b* as 7.422 (the mean of the estimated slope parameters from the HIV gp140 data), and *d* as 0.01. We assume these values to be constant between curves. The values for the point of maximum growth, *c*, were set from -18 to -9, increasing by 0.005. We then calculated the actual values of absorbance using Eq 1 and added a random normal error to represent the background noise. The mean of the random error was 0 and the standard deviation was 0.05004, which was based on the standard deviation of the residuals from model fitting of the 1027 samples in the HIV gp140 binding dataset [4]. We then estimated the PMG, the AS, and the ET using the methods described above for each simulated sample and compared these values to the simulated PMG.

### Statistical power comparisons

Statistical power is the ability to detect a true difference and is often used as a criterion for comparing methods. Having higher power at a fixed significance level indicates a better ability to detect a true difference; therefore, a more desirable method. To compare statistical power associated with the PMG, ET, and AS, we simulated two groups of data with 10 samples in each group (20 total samples). For one group, a set of point of maximum growth (PMG), -16.0595, -15.0959, -13.0959, and -11.5959, were used to represent different scenarios that may appear in real data: two where the measured data cover the PMG, one where the PMG is at the maximum value of the measured data, and one where the PMG is outside of the range of the measured data. These PMGs are constrained within the range for successful sigmoidal curve fitting. Data for the comparison group were simulated using the same settings, except that the PMG differs by values from 0 to 0.75 with an increment of 0.05. PMG, AS, and the ET were estimated for all 20 samples. A t test was then performed to test for differences between the two groups for each of the three methods with a significance level of 0.05. The simulation and testing procedure was repeated 2,000 times for estimating power.

Variations of this simulation were performed by doubling and halving the standard deviation of the random error added. The sample sizes were also varied from 10 to 3 (total sample size from 20 to 6). To examine the impact of heterogeneous variances, we also changed the variance of the random error linearly and quadratically dependent on the absorbance in our simulations. This would result in increasing variance as absorbance increases. We used the formula *y*^*α*^*σ*^2^, where *y* is the simulated absorbance, *α* = 1 for the linear variance, and *α* = 2 for the quadratic variance [10].

Comparison with the AUC method was conducted for three scenarios, 1) with data covering the whole sigmoidal curve, 2) with data located only at the lower tail, and 3) with data located only at the upper tail. Quadratic curves were estimated with the *nlsLM* function.

## Results

### Sigmoidal models for ELISA data

**Fig 2:**
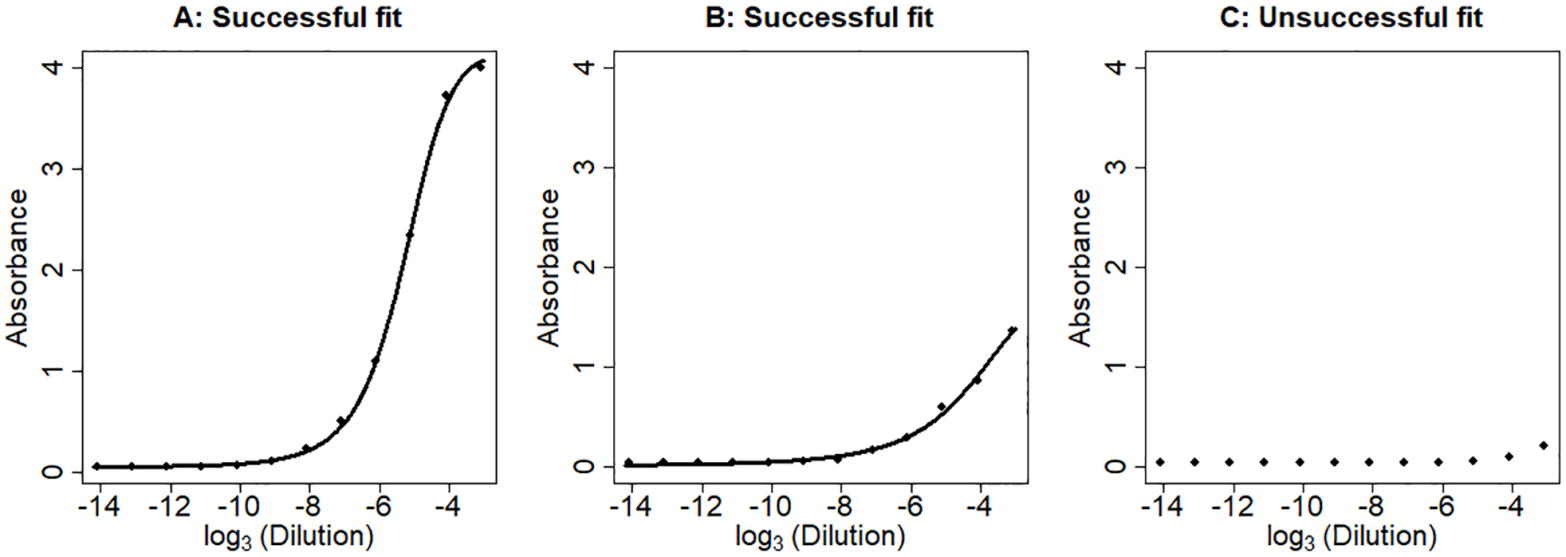
Examples of successful and unsuccessful sigmoidal curve fitting. **A**. Successful sigmoidal curve fit where the data cover the estimated point of maximum growth (PMG). **B**. Successful sigmoidal curve fit even though data are not available for the estimated PMG. **C**. Unsuccessful sigmoidal curve fit due to the lack of informative data for fitting the curve. Line, fitted sigmoidal curve.

Sigmoidal models are often used to model ELISA data. To determine whether there are differences in absorbance (and thus amount of analyte) between samples, sigmoidal curves are compared for differences. The point of maximum growth (PMG) is the parameter of the sigmoidal curve most relevant to the analyte concentration. We estimated this parameter in the HIV gp140 binding ELISA dataset [4]. We were able to fit a sigmoidal curve for 1027 samples out of the total 2879 samples recorded. For the remaining samples, fitting the sigmoidal curve was not possible because the data were only located at the tails of the sigmoidal curves (Fig 2).

### Absorbance summation is highly correlated with the estimated point of maximum growth point

**Fig 3:**
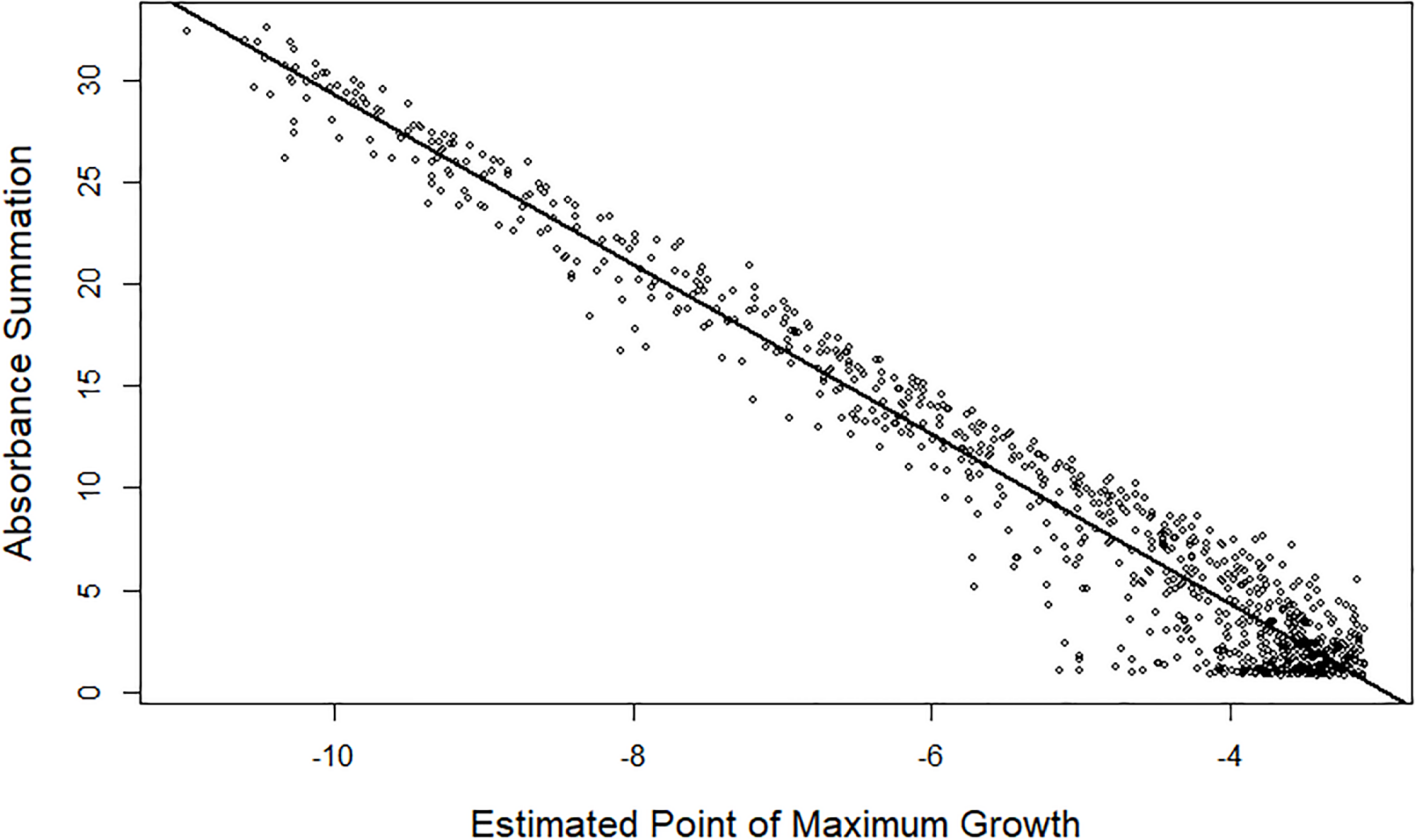
Linear relationship between the absorbance summation (AS) and estimated point of maximum growth (PMG). For observations with an estimated PMG within the observable concentrations, the AS and the PMG have a clear negative correlation. The straight line is the regression line.

We postulated that the absorbance summation (AS) is a good proxy to the analyte concentration. To test this hypothesis, we compared the AS with the estimated PMG for the 1027 samples where the PMG was estimable. A strong and clear linear relationship was observed (Fig 3). As the PMG increases, the AS decreases, which is expected because all absorbance values are smaller as the PMG increases (Fig 2). The correlation between the estimated PMG and the AS is -0.978. Also seen is the increase in variation as the PMG increases, which indicates that estimating PMG past the measured data limit decreases the accuracy.

### In simulations, absorbance summation is better correlated with simulated point of maximum growth than its estimate or endpoint titer

Using the 4-parameter sigmoidal model, we compared the estimated PMG, ET, and AS by simulating ELISA absorbance readings based on the estimates in the HIV gp140 binding dataset. We set equidistant log dilution (-24.0959 to -13.0959) and PMG from -18 to -9 increasing by 0.005. We also added a homogenous random normal error with mean 0 and standard deviation of 0.05004. The choice of constant variance is based on the observation that curve fitting residuals have a roughly constant variance along the fitted curve (S1 Fig).

**Fig 4:**
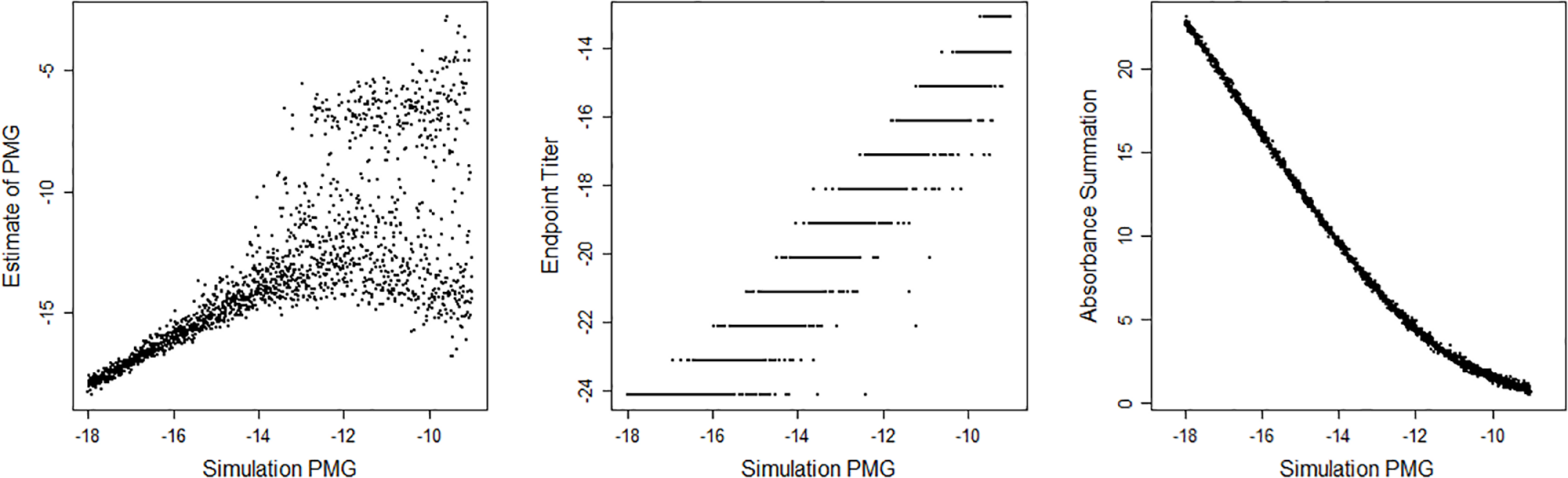
Relationship between the three proxies and simulated PMG. Sigmoidal curves are simulated with different point of maximum growth (PMG). Each estimated proxy is plotted against the simulatied PMG. The estimated PMG works well for values where there is more data surrounding the PMG, but variance increases when the available data are not near the PMG. The endpoint titer method results in discrete values and has a weaker relationship with the actual PMG. The absorbance summation has a strong relationship with the true PMG.

With simulated data, we fit sigmoidal curves and estimated the PMG, ET, and AS. Comparing each of the three proxies with the simulated value of the PMG revealed that even for extreme values AS has the smallest variation (Fig 4). A mathematical justification for the small variation of AS is described in S1 Appendix. The estimation of the PMG works well for about one-third of the simulations, but falls apart when the simulated PMG increases. The limit of PMG estimation is the final log dilution measured at -13.0959, beyond which the estimated PMG does not have any correlation with the simulated PMG. The ET method lacks continuity, as expected, and it also has more variability than the AS method. It is important to keep in mind that we do not need an actual estimate for PMG, we only need a proxy for which we can conduct statistical analyses to infer analyte concentration differences.

### In sample group comparisons, AS has more statistical power than PMG and ET

To compare the ability of the three methods for detecting differences among sigmoidal curves, we compared the statistical power of the three methods in testing groups of samples for difference. We used the 4-parameter sigmoidal model and simulated two groups of samples each with a sample size of 10. We varied the PMG in the simulations so that some are covered by the data and some are not (S2 Fig), and we introduced a set of PMG differences between the two groups, which represents a difference in analyte concentrations. We also varied the residual variance along the sigmoidal curve by setting the variance linearly or quadratically depending on the simulated value on the curve [11]. A two-sample t test was used to compare the groups for differences and to assess the power.

**Fig 5:**
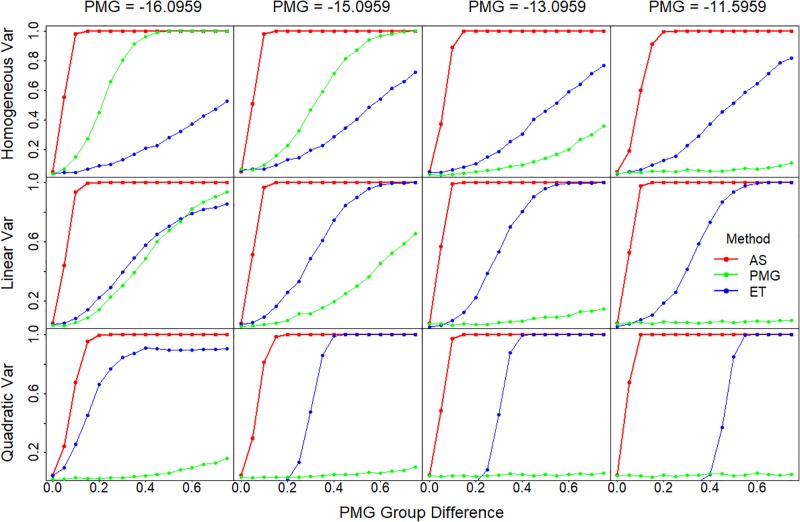
Statistical power comparison among the three methods. Statistical power is plotted against the PMG differences between the two comparing groups. Sigmoidal curves are simulated with different PMG, which range from within (-16.0959 and -15.0959) to outside of the measurements (-13.0959 and -11.5959), as shown in the panel columns. The error variance is either constant or changing along the curve with a linear or quadratic relationship with the values on the curve, which are shown in different panel rows. Statistical power is calculated based on the two group t test. Var, variance.

The AS method proved the most powerful in all the scenarios examined (Fig 5). AS has high power regardless of the settings of PMG and variance structure, whereas the powers of ET and PMG methods are highly dependent on the settings of PMG and the variance structure.

The PMG method performs reasonably well when variance is constant and when the simulated PMGs are small; *i*.*e*., well covered by data. Power decreases dramatically as variance changes or the PMG increases and the data points shift toward the lower tail of the sigmoidal curve. This has been seen in other research as well [3].

The ET method behaves largely opposite to the PMG method. Its power increases as PMG increases, which is related to the fact that data are more concentrated at the lower tail where the ET is estimated. Due to the fact that ET is estimated at the lower tail where the variance is smaller, ET power increases as the variance increases along the sigmoidal curves. Interestingly, ET has no power for small differences in PMGs, but has a sudden increase for quadratic variance. Further examination of these simulations showed that the variance of the mean differences between the two comparing sigmoidal curves starts extremely low or even at zero, and then jumps up when the curve moves to a position where the cutoff is between titers (S3 Fig).

We then examined the influences of the variance of the absorbance readings and sample size on the comparison of these three methods. When variance doubled or halved, the power of all three methods increases or decreases, respectively, as expected. The pattern observed in Fig 5 continues to apply. Large variance diminishes the power of PMG and ET quickly, while the AS power is just slightly affected (S4 Fig). For decreased sample size (n = 3), the effect on the statistical power is similar to that of doubled variance. These results indicate that both the ET and the PMG methods are heavily reliant on the consistency of measurements and reduced error in the absorbance readings. The greater the ‘noise’ in the readings, the greater the difficulty in detecting a difference between samples.

### AS has similar power to AUC method in sample group comparisons

**Fig 6:**
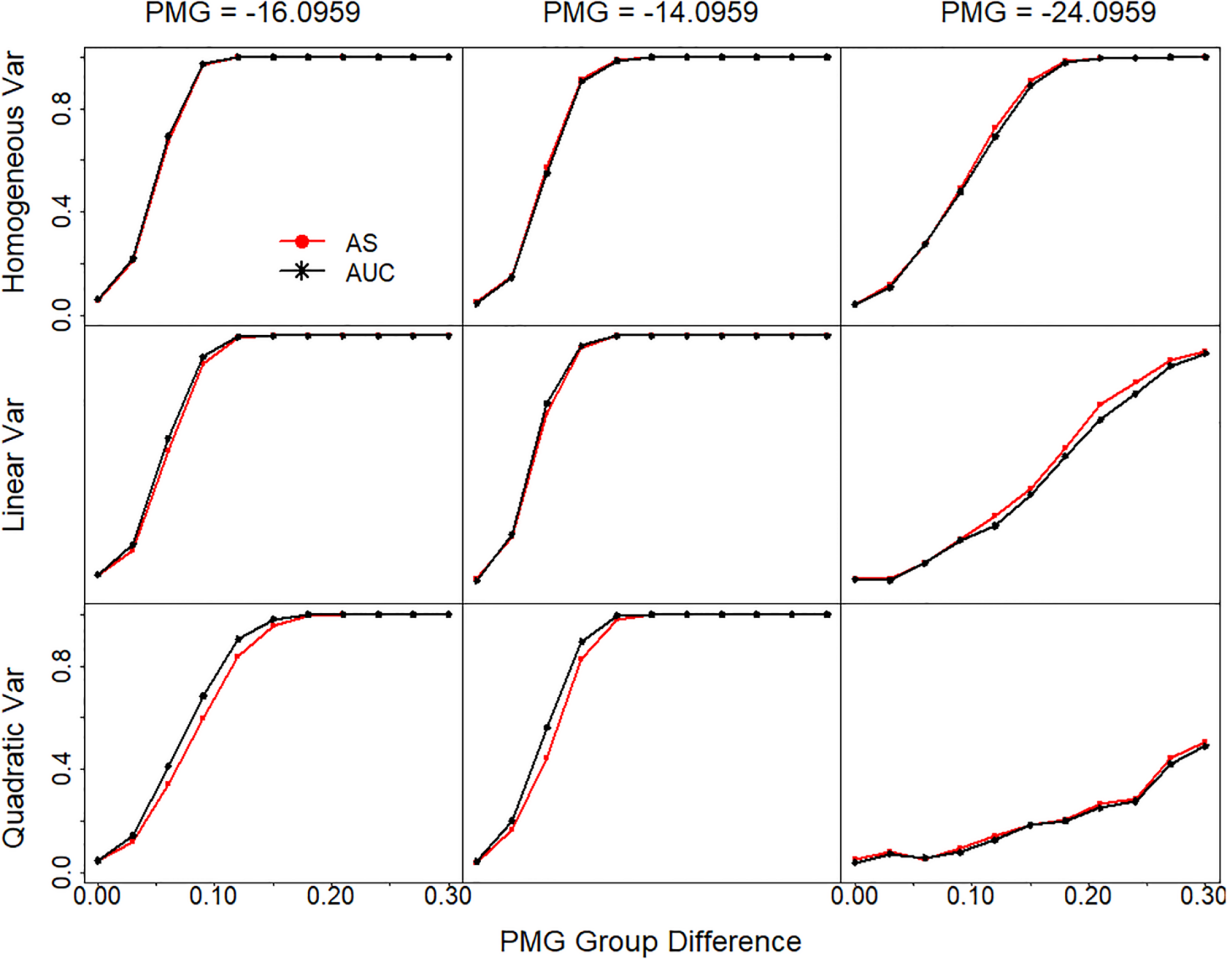
Statistical power comparison between AS and AUC. Statistical power is plotted against the PMG differences between the two comparing groups. Sigmoidal curves are simulated with different PMG (-16.0959, -.0959 and -24.0959 for the three panel columns). The variance for each measurement of the simulated curve is either constant or changing along the curve with a linear or quadratic relationship with the measurement values on the curve in the three panel rows. Statistical power is calculated based on the two group t test. AUC is estimated based on quadratic curve fitting and integration. Var, variance.

Absorbance summation is an approximation of the area under the sigmoidal curve. Recently Yu et. al. [12] proposed the use of the area under the curve (AUC) for the analysis of similar data. Their method first estimates the parameters in a curve, and then integrates the curve from the lowest concentration to the highest concentration. Since the 4-parameter sigmoidal model we used here and the 5-parameter model examined by Yu et. al. are not integrable and require numeric approximation, they recommend the use of a quadratic curve for approximation. To compare our AS method and the AUC method, we fitted a quadratic curve to our sigmoidal simulations and compared the statistical power in two-group comparisons. Although the quadratic curve does not fit the data properly, especially at the upper and lower asymptotes (S5 Fig), the results showed that the two methods have very similar power across different PMG and variance settings (Fig 6). The AUC method has slightly better power when observed data cover the PMG or are mostly located at the lower tail (PMG = -16.0959 and PMG = -14.0959), while AS method has slightly better power when data are mostly located at the upper tail of the sigmoidal curve (PMG = -24.0959).

## Discussion

The advantages of the AS method are that it is both statistically sound and simple to apply. We propose that absorbance summation can serve as a simpler, and thus better, proxy for testing the difference in concentrations of an analyte than other commonly used methods. Our empirical and simulation studies show that that absorbance summation is superior to endpoint titer and sigmoidal curve estimation for testing differences between ELISA dilution curves. Although this study has focused on ELISA, the AS method that we propose here is not limited to ELISA data. It can be applied to any collection of data with a sigmoidal curve for comparing PMGs. Potential applications include growth curves, dose response curves, etc. It will be interesting to examine the application of this method in these other contexts.

Curve fitting and associated PMG estimation, while ideal for obtaining information regarding the curve, is not always possible. Additionally, the complex computations required by this method make it difficult for researchers without access or ability to perform those computations. ET is a simple method that anyone can do, but it has inherent flaws. Since the resulting data of ET method is categorical, it violates the assumption of normality (and thus continuity) of some most commonly used statistical tests, such as the t test. As a result, this method is not desirable, particularly when the variance is not homogeneous. Finally, the method heavily relies on having small variance in measurements. The AUC method is as powerful as the AS method. However, it still involves formal model fitting and integration, which increases complexity. In addition, the parameters of quadratic curve do not have biological meanings.

A potential issue with the AS method occurs when data is missing at certain dilutions. For example, a missing absorbance value due to operator or machine error will result in a summation smaller than expected for that sample. Imputing a value for the missing absorbance for that sample, or removing the absorbance values for that dilution from the other samples can rectify this issue. One potential imputation method would be using the average absorbance for that dilution from other samples or by fitting a sigmoidal model to estimate if possible. However, any imputation will increase complexity of the method. Moreover, imputing or removing data are both clearly less than optimal practices and carry the risk of affecting the reproducibility of the data. The AUC method is less sensitive to sporadic missing data, and it can serve as the alternative when data is missing and the investigators have the ability to perform the computation.

Similar to AUC, the AS method does not work well for comparing curves with same PMG but different slopes at the PMG. Due to the symmetric nature of the sigmoidal curve, the AUCs are the same for curves with same PMG but different slopes. Fortunately, in most cases the same proteins are compared for different concentrations across groups so the slopes should be constant. In the case of matrix effects, the sigmoidal curve may not be symmetric and a 5-parameter sigmoidal model with an asymmetry parameter may be appropriate for fitting such data [13]. We believe our method would be able to overcome this asymmetry and identify changes in the point of inflection if the asymmetry parameter is fixed or slightly varying. How to address matrix effect will be an interesting topic for future research.

In summary, we have developed a simple, but powerful, method of absorbance summation that offers a means to compare data coming from sigmoidal curves. Both AS and AUC appear superior to endpoint titer and sigmoidal curve estimation for testing differences between ELISA dilution curves, especially when there is increased variance or the observed data lie at the lower end of the sigmoidal curve. When compared to the complexity of AUC, AS offers the benefits of simplicity. When using this method, however, the operator should focus on obtaining a complete data set, as the sporadic missing of data can complicate the analysis.

## Supporting information

S1 Appendix Mathematical examination of the relationship between absorbance summation and the point of maximum growth. (DOCX)

S1 Fig Stable variance across the range of the predicted absorbance. With the exception of the extremes where certain actual absorbance values are invalid (below 0 and above 4), the variances of the residuals do not vary. Red solid line, local mean; blue solid line, local one standard deviation; pink dashed line, local two standard deviation.
(DOCX)

S2 Fig Sigmoidal curves used in the simulations. Data were simulated within the two vertical blue lines. Red starts on the line indicate the point of maximum growth (PMG).
(DOCX)

S3 Fig Comparison of the mean difference and the mean standard deviation for the three variance assumptions. When the variance increases with the fitted absorbance values, the variance is smaller for the area where the endpoint titer is selected (absorbance below 0.2). The smaller variance means that the same titer is selected for the ET repeatedly and then jumps when the difference is sufficient to the next titer. This results in the mean difference between samples increasing nonlinearly. Additionally, the variance is low or even 0 and then increases when the transition to the next titer is occurring resulting in a wave pattern.
(DOCX)

S4 Fig Statistical power comparison for changes of variance and sample size. The simulations are the same as those for Fig 4, except for the noted changes in the figure.
(DOCX)

S5 Fig Quadratic model fit to data simulated with 4-parameter sigmoidal model. The green line is the fitted sigmoidal curve and the black curve is the fitted quadratic curve.
(DOCX)

S1 Data ELISA HIV gp140 binding data. (CSV)
