# Supplementary material for: Absorbance summation: A novel approach for analyzing high-throughput ELISA data in the absence of a standard
Source: PLoS One. 2018 Jun 8;13(6):e0198528. doi: 10.1371/journal.pone.0198528 (PMC5993274; doi:10.1371/journal.pone.0198528)
Supplement: S1 Appendix — (DOCX) [file pone.0198528.s001.docx]

While we use the 4-parameter sigmoidal model for simulations and all other analyses, here we use a 3-parameter sigmoidal model for examining the theoretical relationship between the AS and PMG. The 3-parameter model can be obtained from the 4-parameter model by setting $d=0$and re-parameterizing $x$, $c$, and $b$ using a log transformation.

The summation of the discrete points used in our AS method is a Riemann sum approximation of the integrated area under the sigmoidal curve. To examine the theoretical relationship between the AS and PMG, we look at the relationship between the integrated area under the curve and the parameter for the PMG, $c$. We also graphically examine the nature of the relationship between this integration and the point of maximum growth (**Figure A1**). We find that between the highest dilution, $h$, and the lowest dilution, $l$, $c$ (PMG) has an approximately linear relationship with the integral (**Figure A1**). This holds for various values of the other parameters in a 4 parameter sigmoidal curve (**Figure A2**). This suggests that as the PMG increases, we expect AS to decrease linearly when the PMG is within our measured values.

$$\int_{l}^{h} f\left( x \right)=\int_{l}^{h} \frac{a}{1+e^{-b\left( x-c \right)}} dx$$

$$=\int_{l}^{h} \frac{a}{1+e^{-bx+bc}} dx$$

$$=\int_{l}^{h} \frac{a}{1+e^{-bx}e^{bc}} dx$$

$$=\int_{l}^{h} \frac{a}{1+\frac{e^{bc}}{e^{bx}}} dx$$

$$=\int_{l}^{h} \frac{ae^{bx}}{e^{bx}+e^{bc}} dx$$

$$=\frac{a}{b}\ln\left( e^{bx}+e^{bc} \right)\left| {h \atop l} \right.$$

$$=\frac{a}{b}\ln\left( e^{bh}+e^{bc} \right)-\frac{a}{b}\ln\left( e^{bl}+e^{bc} \right)$$

$$=\frac{a}{b}\ln\left( \frac{e^{bh}+e^{bc}}{e^{bl}+e^{bc}} \right)$$


**Figure A1. The theoretical relationship between the summation and the point of maximum growth when using the 3-parameter sigmoidal model**. The lower bound of where is being measured is marked as “*l*” and the upper bound is marked as “*h*” both in blue. The point of maximum growth (*c*) is scaled to be log3. The values for *a*, *b*, *l*, and *h* were all from the average values from the real data.

**Figure A2. The relationship between the point of maximum growth and the summation**. This relationship is approximately linear between the lowest dilution (*l*) and the highest dilution (*h*) measured. In extreme cases where *l* and *h* are very close, the relationship is slightly more sigmoidal.
