# Supplementary material for: Absorbance summation: A novel approach for analyzing high-throughput ELISA data in the absence of a standard
Source: PLoS One. 2018 Jun 8;13(6):e0198528. doi: 10.1371/journal.pone.0198528 (PMC5993274; doi:10.1371/journal.pone.0198528)
Supplement: S1 Fig — With the exception of the extremes where certain actual absorbance values are invalid (below 0 and above 4), the variances of the residuals do not vary. Red solid line, local mean; blue solid line, local one standard deviation; pink dashed line, local two standard deviation. (DOCX) [file pone.0198528.s002.docx]

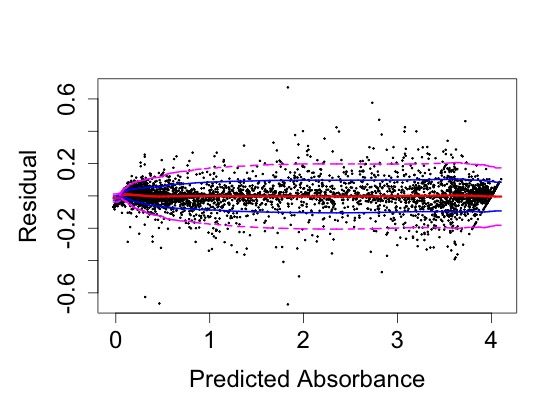


**S1 Fig. Stable variance across the range of the predicted absorbance**. With the exception of the extremes where certain actual absorbance values are invalid (below 0 and above 4), the variances of the residuals do not vary. Red solid line, local mean; blue solid line, local one standard deviation; pink dashed line, local two standard deviation.
