# Supplementary material for: Absorbance summation: A novel approach for analyzing high-throughput ELISA data in the absence of a standard
Source: PLoS One. 2018 Jun 8;13(6):e0198528. doi: 10.1371/journal.pone.0198528 (PMC5993274; doi:10.1371/journal.pone.0198528)
Supplement: S2 Fig — Data were simulated within the two vertical blue lines. Red starts on the line indicate the point of maximum growth (PMG). (DOCX) [file pone.0198528.s003.docx]

**S2 Fig. Sigmoidal curves used in the simulations.** Data were simulated within the two vertical blue lines. Red starts on the line indicate the point of maximum growth (PMG).
