# Supplementary material for: Absorbance summation: A novel approach for analyzing high-throughput ELISA data in the absence of a standard
Source: PLoS One. 2018 Jun 8;13(6):e0198528. doi: 10.1371/journal.pone.0198528 (PMC5993274; doi:10.1371/journal.pone.0198528)
Supplement: S3 Fig — When the variance increases with the fitted absorbance values, the variance is smaller for the area where the endpoint titer is selected (absorbance below 0.2). The smaller variance means that the same titer is selected for the ET repeatedly and then jumps when the difference is sufficient to the next titer. This results in the mean difference between samples increasing nonlinearly. Additionally, the variance is low or even 0 and then increases when the transition to the next titer is occurring resulting in a wave pattern. (DOCX) [file pone.0198528.s004.docx]

**S3 Fig. Comparison of the mean difference and the mean standard deviation for the three variance assumptions.** When the variance increases with the fitted absorbance values, the variance is smaller for the area where the endpoint titer is selected (absorbance below 0.2). The smaller variance means that the same titer is selected for the ET repeatedly and then jumps when the difference is sufficient to the next titer. This results in the mean difference between samples increasing nonlinearly. Additionally, the variance is low or even 0 and then increases when the transition to the next titer is occurring resulting in a wave pattern.
