# Supplementary material for: Absorbance summation: A novel approach for analyzing high-throughput ELISA data in the absence of a standard
Source: PLoS One. 2018 Jun 8;13(6):e0198528. doi: 10.1371/journal.pone.0198528 (PMC5993274; doi:10.1371/journal.pone.0198528)
Supplement: S4 Fig — The simulations are the same as those for Fig 4, except for the noted changes in the figure. (DOCX) [file pone.0198528.s005.docx]

**S4 Fig. Statistical Power comparison for changes of variance and sample size.** The simulations are the same as those for **Fig 4,** except for the noted changes in the figure.
