# Supplementary material for: Absorbance summation: A novel approach for analyzing high-throughput ELISA data in the absence of a standard
Source: PLoS One. 2018 Jun 8;13(6):e0198528. doi: 10.1371/journal.pone.0198528 (PMC5993274; doi:10.1371/journal.pone.0198528)
Supplement: S5 Fig — The green line is the fitted sigmoidal curve and the black curve is the fitted quadratic curve. (DOCX) [file pone.0198528.s006.docx]

**S5 Fig. Quadratic model fit to data simulated with 4-parameter sigmoidal model.** The green line is the fitted sigmoidal curve and the black curve is the fitted quadratic curve.
